# Supplementary material for: Identification of photoperiod-induced specific miRNAs in the adrenal glands of Sunite sheep (Ovis aries)
Source: Front Vet Sci. 2022 Jul 22;9:888207. doi: 10.3389/fvets.2022.888207 (PMC9354845; doi:10.3389/fvets.2022.888207)
Supplement: Supplementary file 1 [file Data_Sheet_1.ZIP › Supplementary file/miRDeep2 file.zip!/miRDeep2 file/Novel_Structure/LP42A2/Novel_358.pdf]

Provisional ID	:	NC_019458.2_1361
Score total	:	2.3
Score for star read(s)	:	-1.3
Score for read counts	:	0
Score for mfe	:	2
Score for randfold	:	1.6
Score for cons. seed	:	
Total read count	:	202
Mature read count	:	172
Loop read count	:	0
Star read count	:	30

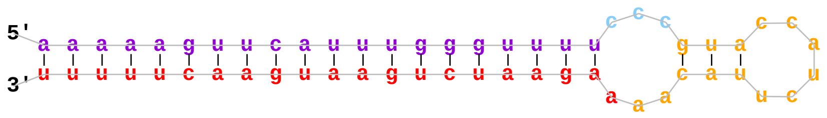

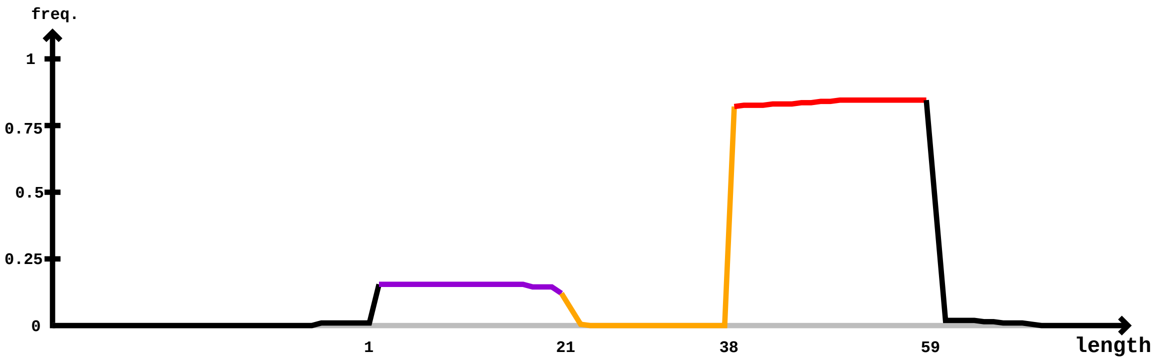


```
                                 Star                                    Mature
5'- caaaaugaaugaacuaugauauugcguuggccaaaaaguucauuuggguuuucccguaccaucuuacaaaagaaucugaaugaacuuuuuggccaacccaauacauucau   -3'    obs
    caaaaugaaugaacuaugauauugcguuggccaaaaaguucauuuggguuuucccguaccaucuuacaaaagaaucugaaugaacuuuuuggccaacccaauacauucau          exp
    ....(((((((........(((((.(((((((((((((((((((((((((((...(((......)))...))))))))))))))))))))))))))).))))))))))))   reads     mm        sample
    ..........................uuggGcaaaaaguucauuuggg..............................................................   2         1         seq
    ................................aaaaaguucGuuuggguuu...........................................................   5         1         seq
    ................................aaaaaguucGuuuggguuuu..........................................................   24        1         seq
    ................................aaaaaguucauuuggguuuucU........................................................   1         1         seq
    .....................................................................aaAaaucugaaugaacuuuuu....................   111       1         seq
    .....................................................................aaAaaucugaaugaacuuuuug...................   59        1         seq
    ......................................................................agaaucugaaugaacuuuuu....................   1         0         seq
    .........................................................................auUugaaugaacuuuuuggcca...............   1         1         seq
    ............................................................................uAaaugaacuuuuuggccaac.............   1         1         seq
    ..............................................................................aaugaacuuuuuggccaaccca..........   1         0         seq
    ................................................................................ugaacuuuuuggccaacccaa.........   1         0         seq
```
